# The lesser trochanter index: detection of increased femoral version in patients with symptomatic hip disease

**DOI:** 10.1186/s13018-026-07084-9

**Published:** 2026-07-21

**Authors:** Klaudia Kaczmarek, Andreas Nitsch, Feras Kasabji, Georgi I. Wassilew, Jens Goronzy, Maximilian Fischer

**Affiliations:** Center for Orthopaedics, Trauma Surgery and Rehabilitation Medicine, University Medicine Greifswald, Greifswald, Germany

**Keywords:** Developmental dysplasia of the hip, Femoroacetabular impingement, Acetabular retroversion, Femoral version, Lesser trochanter index, Hip preservation surgery

## Abstract

**Background:**

Femoral version (FV) affects hip biomechanics and postoperative outcomes in developmental dysplasia of the hip (DDH) as well as femoroacetabular impingement (FAI). However, its assessment depends on advanced imaging modalities such as CT and MRI with limited clinical utility for cost-effective screening of FV. This study aimed to implement and test the diagnostic accuracy of the lesser trochanter index (LTI) as an indicator for increased femoral FV on pelvic radiographs in patients with symptomatic DDH and FAI.

**Methods:**

This prospective, diagnostic cohort study included symptomatic patients with DDH (*n* = 194 patients) and FAI (*n* = 22 patients) undergoing hip preservation surgery. Patients underwent a standardized protocol of anteroposterior pelvic radiographs and magnetic resonance imaging (MRI) of the pelvis, hip and lower extremity. FV was measured in accordance to the Murphy method. The lesser trochanter morphology on pelvic radiographs was used to calculate the lesser trochanter index (LTI). It was predefined as positive to detect increased FV (>25/30° Murphy) with a cut-off value of > 1.5.

**Results:**

A total of 409 hips in 216 patients were included. Patients with DDH were significant older (30.7 vs. 25.5, *p* = 0.008), were more frequently female (77.3% vs. 34.8, *p* < 0.001) and had a significant higher femoral version compared to FAI (28 vs. 21.8, *p* = 0.013). The LTI > 1.5 showed a high sensitivity (25°/30° FV − 90.9/93.1%), but a low specifity (25°/30° FV − 20.3/18.9%) to detect increased FV. The negative predictive value remained moderate (25°/30° FV − 65.5/81%), while the LTI has only a weak positive predictive value (25°/30° FV − 57.5/42.7%).

**Conclusion:**

The LTI represents a clinical useful and widely available indicator of increased FV and can optimize the use of subsequent MRI and CT assessment. However, due to its moderate diagnostic accuracy, the LTI is more a rule- out test, than a diagnostic marker of increased FV and cannot replace advanced imaging modalities.

## Introduction

Hip preservation is primarily guided by three major pathomechanical characteristics including femoral version (FV), joint instability and femoroacetabular impingement (FAI). In this respect, FV has been connected to both instability and impingement while influencing the preoperative diagnostics and ultimately the surgical postoperative outcomes [1]. A high prevalence of femoral malversion has been shown in symptomatic patient cohorts with developmental dysplasia of the hip (DDH) as well as FAI [2, 3]. Furthermore, the surgical correction of femoral malversion in open and arthroscopic hip preserving interventions has been connected to improved postoperative outcomes compared to isolated HD and FAI therapies [4, 5]. Consequently, not addressing femoral malversion can potentially result in an increased risk of poor outcomes and therapeutic failure [6, 7].

Thus, the preoperative diagnostic of pathological femoral version is crucial to maintain the hip joint function and to anticipate postoperative outcomes. Diagnostics of FV are mainly based on advanced imaging by CT or MRI with a wide range of different measurement techniques [8]. This makes studies on femoral malversion hardly comparable and ultimately leading to a wide range of definitions for femoral malversion [9, 10]. Furthermore, as the number of patients and hip preservation surgeries continues to rise, the longer examination time and higher per- patient diagnostic costs make such advanced imaging modalities less affordable as a screening tools for patients with hip pain [11–13].

Therefore, structural characteristics on widely available diagnostic modalities for hip preservation like pelvic radiographs may be useful to screen for the requirement of additional advanced imaging to detect femoral malversion [14]. In this respect, the lesser trochanter morphology have increasingly been studied as potential marker to detect femoral malversion [15, 16]. Even when Jang et al. recently reported less accuracy of the lesser trochanter profile as a marker for FV in a large-scale CT-based study, the lesser trochanter may serve as an indicator of femoral malversion measured on standardized MRI [17]. Thus, a clinically useful indicator for increased FV on widely available pelvic radiographs may improve patient selection for advanced imaging before undergoing hip preservation surgery.

Consequently, this study aimed to test the diagnostic accuracy of the lesser trochanter index (LTI) on plane pelvic radiographs as an indicator for increased FV. It is hypothesized, that LTI is associated with MRI measurements of FV and could additionally be used as a diagnostic tool to detect increased FV in patients undergoing hip preserving surgery.

## Methods

### Study design

This prospective, diagnostic cohort study included 409 hips in 216 symptomatic patients undergoing hip preservation surgery at one tertiary center. All patients were identified from the institutional hip preservation registry and had a standardized preoperative radiographic assessment including pelvic radiographs and magnetic resonance imaging (MRI) based FV measurement. Only native hip joints without history of pelvic or femoral osteotomy were included, leading to an exclusion of 23 hips with previous pelvic redirectional osteotomy or femoral rotational osteotomy. The patients gave written informed consent prior to inclusion. Ethics approval (BB099/20a) was obtained from the local independent ethics committee of the University Medicine Greifswald according to the World Medical Association Declaration of Helsinki.

### Radiographic assessment and calculation of LTI

Anteroposterior pelvic radiographs were performed in supine position with ankles fixed in 15° internal rotation. DDH was classified using a lateral center-edge angle (LCEA) threshold of < 25° and FAI due to acetabular retroversion with all three signs of acetabular retroversion (crossing- over, posterior wall, sciatic spine sign) positive on anteroposterior pelvic radiographs and a LCEA > 25° [18].

**Fig. 1:**
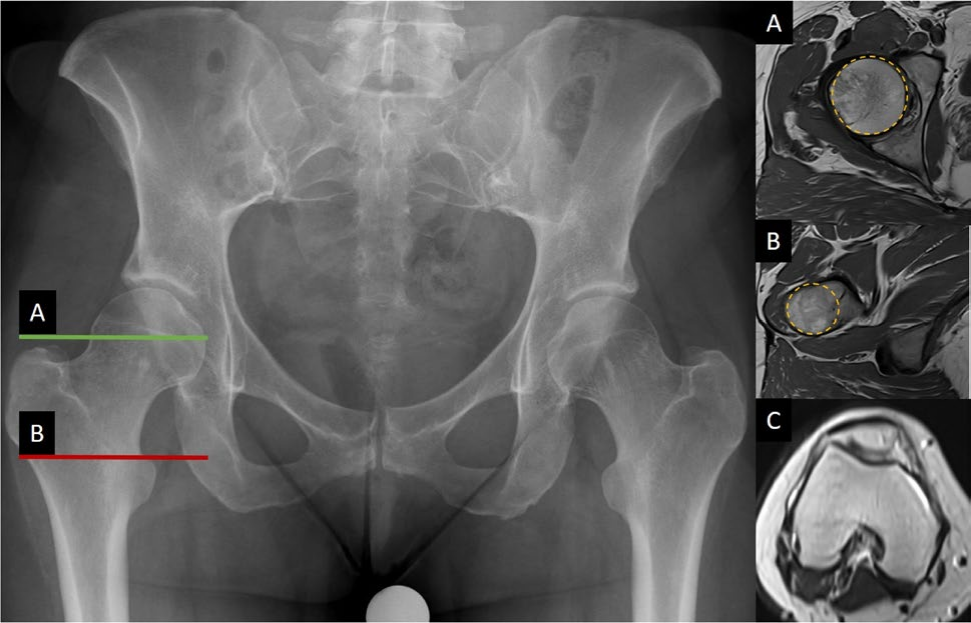
Measurement of femoral version on MRI according to the Murphy method. Representative patient case. **A** Femoral head center **B** base of the femoral neck **C** condylar axis

The FV was measured by MRI with a standardized protocol in accordance to the method described by Murphy [19]. The angle of femoral version was constructed by the femoral head center, femoral neck base and condylar axis (Fig. 1). MRI was performed in a 3T full body scanner (Magnetom VIDA, Siemens, Germany) with ankle fixation to prevent spontaneous rotation during MRI. Only slices in transversal orientation were acquired. The pelvis scan was extended to the distal edge of the lesser trochanter. The knees and the ankle joints were examined applying a T2 turbo spin echo sequence for fast acquisition. The diagnostic accuracy of the LTI to detect increased FV was tested for clinically relevant thresholds of >25° and >30° [20, 21].

**Fig. 2:**
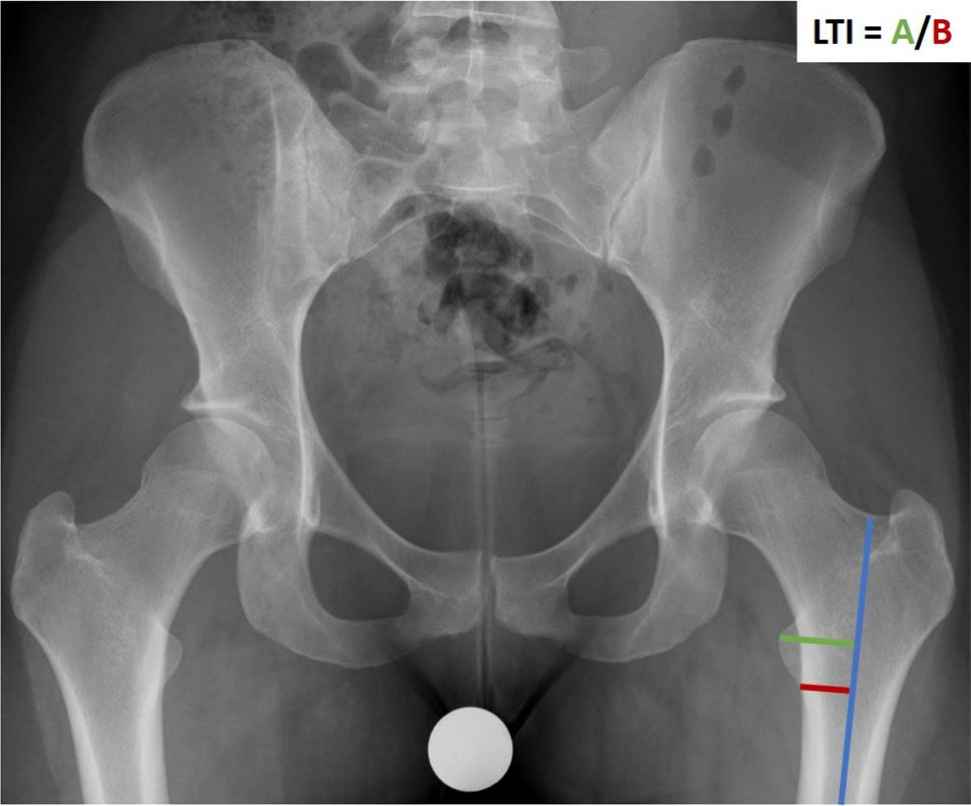
Representative case to calculate the LTI on pelvic radiographs

The LTI was calculated after a standardized stepwise protocol on supine pelvic radiographs. It was predefined as positive for increased FV at values > 1.5 (Fig. 2):

1. Defining the diaphyseal axis of the femur (blue line).
2. Perpendicular to the diaphyseal axis measurement of maximal distance between lesser trochanter prominence and diaphyseal axis (green line - A).
3. Perpendicular to the diaphyseal axis measurement of distance between lesser trochanter basis and diaphyseal axis (red line - B).
4. LTI = A/B.

For validation, LTI on 63 hips were assessed twice by the first author (KK) and additionally by the senior author (MF) for measurement of reliability and reproducibility. The intraclass correlation was 0.95 for inter- rater reliability (left hips – 0.94; right hips 0.95) and intra- rater reliability (left hips – 0.96; right hips 0.94).

### Statistical analysis

Descriptive statistics were used to summarize patient demographics and radiographic parameters. Group comparisons were selected based on assessment of normality and test assumptions. Age and the LTI-index were compared using the Mann–Whitney U test, femoral version was compared using the independent-samples *t*-test, and sex distribution was compared using Fisher’s exact test.

A predefined LTI cut-off value of > 1.5 to detect increased FV was used for the primary analyses based on its clinical applicability including calculation of sensitivity, specifity, positive predictive value, and negative predictive value. Receiver operating characteristic (ROC) curve analysis and the area under the curve (AUC) with 95% confidence interval was performed to evaluate diagnostic accuracy. The statistically optimal LTI cut- off value was determined using the Youden index.

Statistical analysis was conducted using Python 3.11 (pandas, scipy.stats, seaborn, and statsmodels). A two-sided p-value < 0.05 was considered statistically significant.

## Results

Overall, 194 patients (365 hips) with DDH and 22 patients (44 hips) with FAI were included in the final analysis. Patient demographics showed significant differences regarding age, sex distribution and mean FV (Table 1). The DDH group were significant older (30.7 vs. 25.5, *p* = 0.008), more frequently female (77.3% vs. 34.8, *p* < 0.001) and had a significant higher mean FV compared to the FAI group (28 vs. 21.8, *p* = 0.013) (Table 1).

**Table 1:** Patient characteristics

	DDH (194)	FAI (22)	*p*
Age (years)	30.7 ± 8.4	25.5 ± 5.4	0.008
n (% female)	150 (77.3%)	8 (34.8%)	< 0.001
Femoral version (°)	28.0 ± 12.8	21.8 ± 10.3	0.013
LTI	1.68 ± 0.15	1.71 ± 0.16	0.454

Mean with Standard deviation, *LTI* Lesser trochanter index

**Fig. 3:**
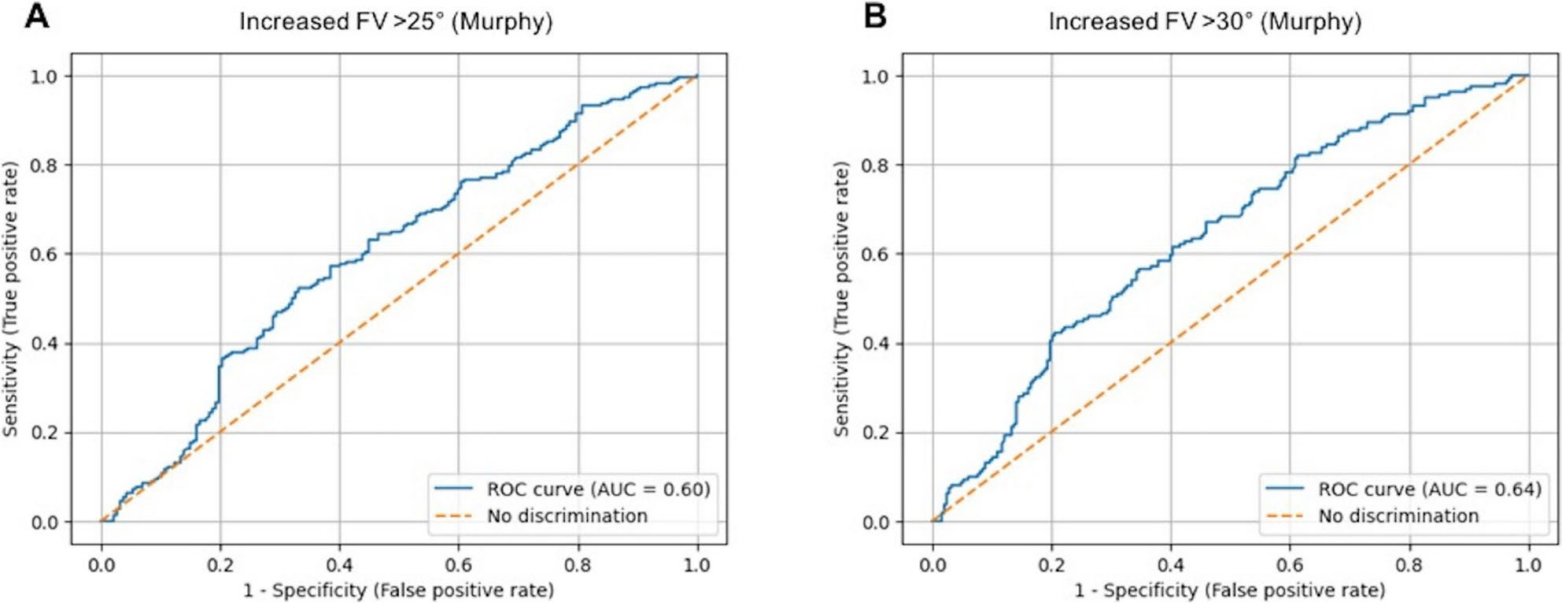
ROC curves for both thresholds of increased FV. **A** Increased FV > 25° (Murphy) **B** Increased FV > 30° (Murphy)

Spearman correlation showed an overall weak correlation between LTI and femoral version (*r* = 0.25, *p* < 0.001). Accordingly, ROC analysis demonstrated a weak discriminatory accuracy of the LTI with an AUC of 0.64 (30° Murphy) and 0.60 (25° Murphy) (Fig. 3A/B).

Defining the LTI with a clinically practical cut-off value > 1.5 to detect increased FV (25/30° Murphy), the sensitivity was 90.9% and 93.1% respectively. The LTI specifity was 20.3% to detect increased FV with a threshold of 25° and 18.9% defining increased FV > 30°. Additionally, LTI had positive predictive value to detect increased FV of 57.5% (25° Murphy) and 42.7% (30° Murphy). The negative predictive value to rule out increased FV was higher when using the 30° threshold to define increased FV (25°/30° Murphy – 65.5%/81%) (Table 2).

**Table 2:** Quality metrics of the lesser trochanter index (LTI)

	LTI - (*n*)	LTI + (*n*)	Sensitivity (%)	Specificity (%)	PPV (%)	NPV (%)
*25° (Murphy)*			90.9	20.3	57.5	65.5
*MRI - (n)*	38	149				
*MRI + (n)*	20	202				
30° (Murphy)			93.1	18.9	42.7	81
*MRI - (n)*	47	201				
*MRI + (n)*	11	150				

*MRI* Positive cut- off > 25/30° (Murphy), *LTI* Positive threshold > 1.5 Mean with Standard deviation, *LTI* Lesser trochanter index, *PPV* Positive predictive value, *NPV *Negative predictive value

Youdens index calculation led only to a marginal clinically- relevant gain in diagnostic performance. The optimal LTI to detect increased FV (> 30° Murphy) was 1.69, yielding a sensitivity of 52% and a specifity of 67%.

## Discussion

The primary aim of the present study was to develop a cost-effective and clinically practical radiographic indicator of increased FV on pelvic radiographs in patients undergoing hip preservation surgery. It was found that a LTI > 1.5 has a high sensitivity and moderate negative predictive value to rule- out increased FV. However, the test accuracy remains not adequate to replace advanced imaging modalities for the definitive diagnosis of increased FV.

Besides testing the diagnostic accuracy of LTI, the present study found a higher mean FV in DDH, which were more frequently female. Comparing this results with the literature, the present study confirms this association between increased FV and hip instability, which has been reported in both asymptomatic as well as symptomatic patient cohorts [21, 22]. Interestingly, even in sex-balanced analysis, increased FV is more frequent in DDH than in FAI patients [23]. Nevertheless, FV must be assessment in each patient with symptomatic DDH as well as FAI since it influences hip function as well as patients’ symptoms [24, 25].

The diagnostic of FV within preoperative patients’ assessment is still a domain of advanced imaging such as CT and MRI [26, 27]. However, pelvic radiographs are the basis of radiographic imaging in every patient with symptomatic hip disease [14]. Consequently, indicators of acetabular retroversion or joint micro- instability assessable on those widely available imaging modality has already been introduced successfully in clinical routine [18, 28]. The data of the current study demonstrate, that similar to those established radiographic markers, lesser trochanter morphology on pelvic radiographs could be used to anticipate FV and allows clinically practical calculation of the LTI.

Nevertheless, in contrast to previous reports, the current data found only a weak association between lesser trochanter morphology and FV [15]. Even when differences in imaging modalities and measurement technique between both studies could explain those discrepancies, the tibial version is a second relevant factor that could influence lesser trochanter morphology on pelvic radiographs even in standardized radiographic protocols with fixed ankles [10, 29]. In this respect, the impact of the overall lower limb alignment could be an explanation for the LTIs limited discriminatory accuracy between normal and increased femoral FV.

Compared to popular indicators of hip instability on pelvic radiographs, the LTI shows similar high sensitivity but an even lower specificity and negative predictive value. For instance, Zimmerer et al. found a sensitivity and specificity of greater than 80° for both FEAR index as well as gothic arch angle to discriminate between stable and unstable hips [28, 30]. Accordingly, even an negative LTI < 1.5 has a limited reliability to exclude increased FV and advanced imaging by CT or MRI is still required. Nevertheless, the LTI could serve as an additional diagnostic sign of increased FV and could be a clinically useful indicator to optimize the use of advanced imaging modalities aiming to reduce the frequency of screening CT or MRI in patients with hip pain.

There are several limitations needing further discussion. First, this study consisted of a selected patient cohort undergoing hip preservation surgery. Only symptomatic hips without osteoarthritis (Tönnis grad < 2) were included and hips with history of pelvic or femoral osteotomy were excluded. Furthermore, the present study was imbalanced towards DDH patients and included only a little number of FAI patients. All of these circumstances may limit the generalizability to other hip pathologies. The present study cohort included a high proportion of patients with increased FV leading to the fact that the diagnostic accuracy of the LTI was tested only for increased FV. Ongoing research may further use the LTI in FAI patient cohorts to define a cut- off value to detect femoral retroversion. Next, given diagnostic nature of this cohort study, the LTI was not correlated with clinical outcomes. Further studies including postoperative outcomes are required to determine the feasibility of the LTI as a preoperative indicator of increased FV affecting advanced imaging utilization and therapy decisions. Finally, although all patients underwent a standardized radiographic protocol, this study was unable to detect the exact location of femoral deformity. The MRI assessment included the proximal femoral morphology, the lesser trochanter, and the knee region, but did not evaluate the femoral diaphysis. Therefore, femoral deformity located in the diaphyseal area may not has been detected, which could interfere with the diagnostic accuracy of the LTI.

## Conclusion

The LTI calculated on pelvic radiographs can optimize the use of CT and MRI diagnostics in patients undergoing hip preserving surgery as a cost- effective and clinically useful indicator of increased FV. Using a cut- off > 1.5, the LTI correctly excludes increased FV (30° Murphy) in four out of five patients. However, the test accuracy of the LTI remains not sufficient to completely replace advanced imaging modalities for the evaluation of increased FV.

## Data Availability

The datasets generated and analyzed in the current study are not publicity available due to data protection regulations. Inquiries for data access can be made to the corresponding author.
